# The global regulator Crc plays a multifaceted role in modulation of type III secretion system in *Pseudomonas aeruginosa*

**DOI:** 10.1002/mbo3.54

**Published:** 2013-01-04

**Authors:** Yi-Hu Dong, Xi-Fen Zhang, Lian-Hui Zhang

**Affiliations:** Institute of Molecular and Cell Biology61 Biopolis Drive, Singapore, 138673

**Keywords:** Crc, exsA, *Pseudomonas aeruginosa*, type III secretion system

## Abstract

The opportunistic pathogen *Pseudomonas aeruginosa* utilizes type III secretion system (T3SS) to translocate effector proteins into eukaryotic host cells that subvert normal host cell functions to the benefit of the pathogen, and results in serious infections. T3SS in *P. aeruginosa* is controlled by a complex system of regulatory mechanisms and signaling pathways. In this study, we described that Crc, an RNA-binding protein, exerts a positive impact on T3SS in *P. aeruginosa*, as evidenced by promoter activity assays of several key T3SS genes, transcriptomics, RT-PCR, and immunoblotting in *crc* mutant. We further demonstrated that the regulatory function of Crc on the T3SS was mediated through the T3SS master regulator ExsA and linked to the Cbr/Crc signaling system. Expression profiling of the *crc* mutant revealed a downregulation of flagship T3SS genes as well as 16 other genes known to regulate T3SS gene expression in *P. aeruginosa*. On the basis of these data, we proposed that Crc may exert multifaceted control on the T3SS through various pathways, which may serve to fine-tune this virulence mechanism in response to environmental changes and nutrient sources.

## Introduction

*Pseudomonas aeruginosa* is a ubiquitous environmental bacterium capable of causing serious and often life-threatening infections in cystic fibrosis patients and immunocompromised humans. This opportunistic pathogen is equipped with a large number of virulence mechanisms and one of the major virulence factors is the type III secretion system (T3SS), which facilitates the delivery/translocation of bacterial effector proteins into eukaryotic host cells to inhibit phagocytosis, resulting in cytotoxicity and tissue destructions in the host (Ghosh 2004; Sato and Frank 2004; Journet et al. 2005; Yahr and Wolfgang 2006).

Regulation of T3SS in *P. aeruginosa* is complicated and it involves both genetic and environmental factors. There are about 43 coordinately regulated genes encoding the type III secretion and translocation machinery, regulatory functions, type III effectors, and effector-specific chaperones (Frank 1997). Expression of the T3SS genes is tightly regulated and under the direct transcriptional control of the master regulator ExsA, a member of the AraC family of transcriptional activators (Yahr and Frank 1994; Hovey and Frank 1995). The activity of ExsA is controlled by interaction with the anti-activator protein ExsD (McCaw et al. 2002), and the ExsD–ExsA interaction can be disrupted by tight binding between the anti-anti-activator ExsC and ExsD (Dasgupta et al. 2004). ExsC activity is in turn controlled by a secreted protein ExsE (Rietsch et al. 2005; Urbanowski et al. 2005). In addition to these T3SS-specific regulatory proteins, other regulators and proteins are also known to affect the expression of T3SS genes. They include, nonexhaustively, the membrane-associated adenylcyclase CyaB; cyclic AMP (cAMP)-binding protein Vfr (Wolfgang et al. 2003); stationary-phase sigma factor RpoS (Hogardt et al. 2004); alginate biosynthesis proteins MucA/AlgU/AlgR (Wu et al. 2004); two-component systems RtsM/RetS and GacA/GacS; RNA-binding protein RsmA (Goodman et al. 2004; Laskowski et al. 2004; Zolfaghar et al. 2005); small proteins PtrB and PtrC (Wu and Jin 2005; Jin et al. 2011); type IV pili biogenesis protein FimL (Whitchurch et al. 2005); transcriptional activator PsrA (Shen et al. 2006); tryptophan synthase TrpAB and tryptophan dioxygenase KynA (Shen et al. 2008); nitrite reductase NirS (van Alst et al. 2009); magnesium transporter MgtE (Anderson et al. 2010); transmembrane protein FlhA (Bange et al. 2010) and cAMP phosphodiesterase CpdA (Fuchs et al. 2010). However, it is not clear how these proteins exert their influence on T3SS.

Expression of *P. aeruginosa* T3SS is also influenced by various environmental conditions, such as calcium depletion and direct contact with host cells or serum (Yahr et al. 1995; Frank 1997). Quorum-sensing signals *N*-butyryl-homoserine lactone (BHL) and Pseudomonas quinolone signal (PQS) as well as other small molecules, such as acetyl-CoA, histidine, and spermidine have also been reported to influence the expression of T3SS genes (Wolfgang et al. 2003; Rietsch et al. 2004; Bleves et al. 2005; Rietsch and Mekalanos 2006; Zhou et al. 2007; Singh et al. 2010). Osmotic stress, metabolic signals, and specific metabolites such as tryptophan catabolites were found to have a profound influence on the expression of T3SS genes, although the molecular regulatory mechanisms linking these metabolite/stresses remains obscure (Aspedon et al. 2006; Shen et al. 2008). It is plausible that *P. aeruginosa* has evolved several complicated regulatory mechanisms and signaling pathways to fine-tune the regulation of T3SS in response to environmental changes.

Crc is a catabolite repression control protein involved in carbon regulation of several pathways in the *Pseudomonas* species (MacGregor et al. 1991, 1996; Morales et al. 2004). Recently, Crc was shown to have RNA-binding properties and it is capable of binding to the A-rich region of target mRNAs to inhibit their translation initiation (Moreno et al. 2007, 2009, 2010; Sonnleitner et al. 2009). In *P. aeruginosa*, the activity of Crc is modulated by a small RNA CrcZ, which is able to bind to and sequester the Crc protein with high affinity and thus relieve repression of Crc targets. The expression of CrcZ is controlled by CbrA/CbrB two-component system in response to different carbon sources (Sonnleitner et al. 2009). In addition to catabolite repression, Crc was found to be central in the regulation of virulence and virulence-related processes, and these include cell motility, biofilm formation, and antibiotic resistance. Inactivation of the *crc* gene impaired swimming, swarming, and twitching motilities, and increased exopolysaccharides production and biofilm formation in *P. aeruginosa* (O'Toole et al. 2000; Linares et al. 2010). The *crc* mutant also exhibited reduced virulence to *Dictyostelium discoideum* and reduced expression of quorum sensing-regulated virulence determinants, such as cyanide, pyoverdine, and elastase, but increased pyocyanin production (Linares et al. 2010; Yeung et al. 2011). Proteomic analysis revealed that three proteins, NirS, GltA, and AceE, which were reported to be involved in T3SS modulation, were differentially expressed in the *crc* mutant and this correlated with a downregulation in the protein levels of the four T3SS secretion proteins, PscN, PopD, PcrV, and ExoS from the *crc* mutant culture supernatants (Linares et al. 2010). Real-time quantitative polymerase chain reaction (RT-qPCR) on *cbrA*, *cbrB*, *crcZ*, and *crc* mutant revealed altered expression of several T3SS genes during *P. aeruginosa* infection of HBE cell line (Yeung et al. 2011). However, it remains unknown how Crc modulates T3SS. Here, we present a detailed analysis and systematic study of Crc on its influence on the T3SS and propose that Crc modulates T3SS through multiple regulatory pathways.

## Materials and Methods

### Bacterial strains, media, and growth conditions

*Pseudomonas aeruginosa* strains used in this study are derived from the prototrophic laboratory strain PAO1. These strains and other bacteria used in this study are listed in Table 1. Bacteria were routinely grown at 37°C in Luria–Bertani broth (LB) or LB medium supplemented with the chelating reagent nitrilotiracetic acid (NTA) at a final concentration of 5.0 mmol/L for induction of T3SS. Mins (Nicas and Iglewski 1984) and BSM (Sonnleitner et al. 2009) minimal medium was also used. Three different carbon sources, mannitol, glucose, and succinate were tested in BSM minimal medium at final concentration of 40 mmol/L and with/without addition of 1 mmol/L spermidine, which induces the transcription of T3SS genes. Antibiotics were used when necessary at the following concentrations: carbenicillin, 300 μg/mL for *P. aeruginosa*, 200 μg/mL for *Escherichia coli*; tetracycline, 100 μg/mL for *P. aeruginosa* and 10 μg/mL for *E. coli*; and gentamicin, 50 μg/mL for *P. aeruginosa* and 5 μg/mL for *E. coli*.

**Table 1:** Bacterial strains and plasmids used in this study^1^

Strain or plasmid	Relevant genotype or phenotype	Source or reference
*Pseudomonas aeruginosa*		
PAO1	Prototrophic laboratory strain	Laboratory collection
PAO1pClacZ	PAO1 with chromosome integration of *exsCEBA* promoter fused with *lacZ*	Zhou et al. (2007)
p39	Transposon mutant with disruption of *crc* gene in PAO1pClacZ	This study
P39(*crc*)	P39 containing pUCP-*crc*	This study
Δ*crc*	PAO1 with in-frame deletion of *crc*	This study
Δ*crc*(*crc*)	Δ*crc* containing pUCP-*crc*	This study
Δ*crc*pClacZ	PAO1pClacZ with in-frame deletion of *crc*	This study
Δ*crc*pClacZ(*crc*)	Δ*crc*pClacZ containing pUCP-*crc*	This study
Δ*cbrB*pClacZ	PAO1pClacZ with in-frame deletion of *cbrB*	This study
ΔCrcZpClacZ	PAO1pClacZ with deletion of CrcZ	This study
*exsA*::Tn	Transposon mutant with disruption of *exsA* gene in PAO1pClacZ	This study
PAO1pTlacZ	PAO1 with chromosome integration of *exoT* promoter fused with *lacZ*	Zhou et al. (2007)
PAO1pDlacZ	PAO1 containing a vector of pME-P*exsD–lacZ*	This study
PAO1pD’-‘lacZ	PAO1 containing a vector of pME-P*exsD*’-‘*lacZ*	This study
PAO1pN’-‘lacZ	PAO1 containing a vector of pME-*PpopN*’-‘*lacZ*	This study
*Escherichia coli*		
DH5α	F^−^ϕ80d*lacZ*ΔM15 *endA1hsdR17* (r_k_^−^ m_k_^−^. *supE44 thi-1 gyrA96* Δ(*lacZYA-argF*)	Gibco
S17-1	*recA pro* (RP4-2Tet::Mu Kan::Tn*7*)	Simon et al. (1983)
Plasmid		
pBT20	Mariner transposon vector; Gm^r^, Ap^r^	Kulasekara et al. (2005)
pUCP19	*E. coli – P. aeruginosa* shuttle vector with the *lac* promoter (P*lac*), Amp^r^/Cb^r^	ATCC 87110
pUCP-crc	pUCP19 containing *crc* under the control of P*lac*	This study
pUCP-exsA	pUCP19 containing *exsA* under the control of P*lac*	This study
pEX18Gm	*P. aeruginosa* gene replacement vector; *sacB*, Gm^r^/Cb^r^	Hoang et al. (1998)
pEX-de *crc*	pEX18Gm carrying the *crc* flanking region with the gene being deleted	This study
pEX-de *cbrB*	pEX18Gm carrying the *cbrB* flanking region with the gene being deleted	This study
pEX-de CrcZ	pEX18Gm carrying the CrcZ flanking region with the gene being deleted	This study
pME6010	pVS1-p15A shuttle vectors	Heeb et al. (2000)
pME2-lacZ	pME6010 carrying a full-length *lacZ*	Dong et al. (2008)
pME-P*exsDlacZ*	pME2-*lacZ* carrying *exsD* promoter in front of *lacZ*	This study
pME-P*exsD*’-‘*lacZ*	P*exsD*’-‘*lacZ* translational fusion	This study
pME-P*popN*’-‘*lacZ*	P*popN*’-‘*lacZ* translational fusion	This study

1 Amp^r^, ampicillin resistant; Cb^r^, carbenicillin resistant; Gm^r^, gentamicin resistant.

### Plasmid and mutant construction

The plasmids used in this study are listed in Table 1. The Mariner transposon carried by plasmid pBT20 (Kulasekara et al. 2005) was used to generate the transposon mutants and the mutants were screened for genes involved in the regulation of *exsCEBA* expression as described previously (Zhou et al. 2007). The genes disrupted by transposon insertion were identified by PCR (Caetano-Annoles 1993). All deletion mutants of *P. aeruginosa* were produced by using pEX18Gm plasmid (Hoang et al. 1998) containing the PCR fragment flanking each gene for in-frame deletion of the internal sequence following the methods described previously (Dong et al. 2008). The deletion mutants were confirmed by PCR.

For complementation of the mutant and in trans expression, the coding regions of the native gene were amplified from PAO1 genomic DNA and the PCR products were cloned at *Hin*dIII and *Bam*H1 sites in the shuttle vector pUCP19 (ATCC 87110). To construct the PAO1pDlacZ reporter, a 568-bp fragment spanning −409 to +159 bp relative to the translational start site of the *exsD* gene was amplified from the genomic DNA of *P. aeruginosa* strain PAO1 by PCR. This fragment contains the engineered *Hin*dIII and *Eco*RI sites. The *Hin*dIII- and *Eco*RI-digested fragments were then ligated into the corresponding sites of pME2-lacZ (Dong et al. 2008). The P*exsD*’-‘*lacZ* and P*popN*’-‘*lacZ* translational fusion reporter plasmids were constructed by using the fragments (−409 to +159 for *exsD* and −243 to +120 for *popN*) amplified from PAO1 genomic DNA and ligated into pME'lacZ translational fusion vector (Heeb et al. 2000). These constructs were introduced into *P. aeruginosa* by electroporation, and the transformants were selected on LB agar plates containing relevant antibiotics.

### Immunoblotting detection

Whole-cell lysate samples were prepared from different bacterial strains grown in LB broth supplemented with NTA to an absorbance (*A*_600_) of ≍1.3–1.5. After sonication, samples were analyzed by 12% SDS–PAGE followed by either InstantBlue (Expedeon, Cambridgeshire, U.K.) staining or transferred to nitrocellulose membranes for detection by immunoblotting with antibodies against ExsA, ExsD (generously provided by Dr. Sylvie Elsen, iRTSV, France), *E. coli* β-galactosidase, and *E. coli* RNA polymerase (RNAP), respectively, as indicated.

### RNA extraction, microarray, RT-PCR, and real-time PCR analysis

Overnight cultures of *P. aeruginosa* strains were diluted in Mins medium and incubated at 37°C until the OD_600_ reached about 1.5. Total RNA samples were then isolated using the RNeasy miniprep kit (Qiagen). Following DNase I (Promega) digestion, RNA samples were quantified and analyzed for purity by agarose gel electrophoresis and by PCR to make sure that no contaminating genomic DNA was present. cDNA was synthesized by using random primers (Invitrogen), SuperScript III (Invitrogen) and biotin-ddUTP according to the protocol from Affymetrix (Santa Clara, CA). Target hybridization, washing, and staining were performed following the manufacturer's instructions on an Affymetrix GeneChip fluidics station. GeneChip arrays were scanned using an Affymetrix probe array scanner. The data were analyzed by using Affymetrix Expression Console Software and the RMA algorithm was used for comparing gene expression between mutant and wild-type strain. Two independent experiments were performed and the results were showed as fold change. Reverse transcription PCR (RT-PCR) was performed using OneStep RT-PCR Kit (Qiagen) and RT-qPCR analysis was carried out by using QuantiTect SYBR Green PCR kit (Qiagen) on the LightCycler® 4.0 system (Roche) according to the manufacturer's instruction.

### Quantitative β-galactosidase assay

For measurement of β-galactosidase activity, *P. aeruginosa lacZ* reporter strains were grown in different media as indicated with shaking at 37°C until reaching an OD_600_ of 1.0–1.5. Bacterial cells were collected and measured for β-galactosidase activity. Results were given as Miller units of β-galactosidase activity per OD_600_.

### Cytotoxicity assay

The cytotoxicity of *P. aeruginosa* strain PAO1 and its *crc* deletion mutant were measured using the Cell Counting Kit-8 (Dojindo Molecular Technologies, Rockville, MD), which is based on the dehydrogenase activity detection in viable cells. Bacterial strains were grown in LB medium with 5.0 mmol/L NTA till an OD_600_ of 1.0. The bacterial cells were washed and diluted in Dulbecco's Modified Eagle Medium and then inoculated into the monolayer of HeLa or A549 cells in a 96-well plate at a multiplicity of infection of 50. Absorbance at 450 nm was measured and the cell viability was calculated according to the manufacturer's protocol.

## Results

### Mutation of the carbon metabolism regulator Crc resulted in reduced transcription of *exsCEBA*

**Figure 1:**
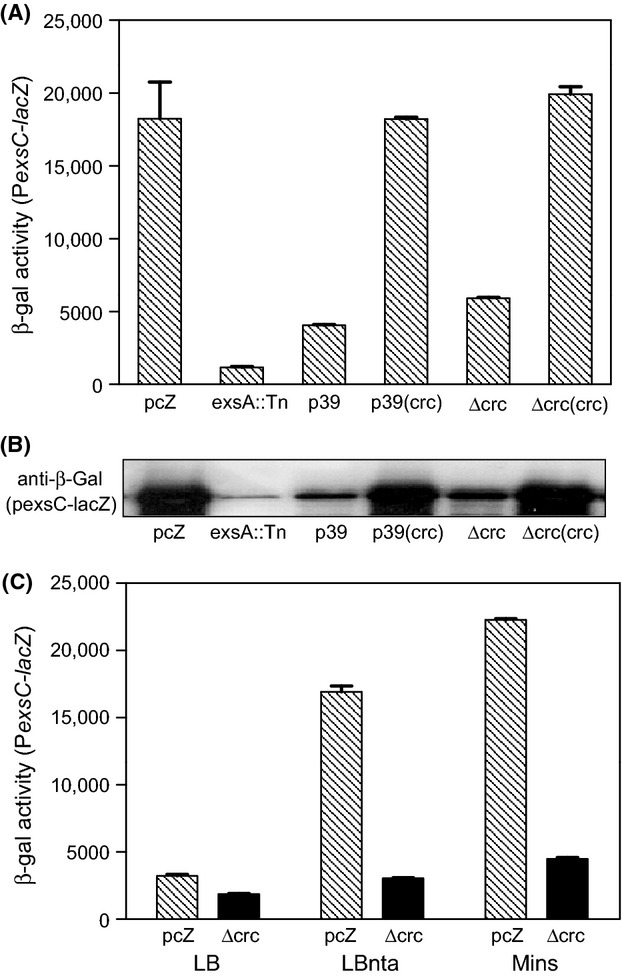
Mutation in the carbon metabolism regulator, Crc, reduced the expression of *exsCEBA*. (A) Transcriptional expression of P*exsC–lacZ* in *crc* transposon mutant (p39) and deletion mutant (Δ*crc*) as well as their corresponding *crc*-complemented strains under type III secretion system-inducing condition. The parental strain PAO1pClacZ (pcZ), which carried a single copy of P*exsC–lacZ* transcriptional reporter gene on its chromosome and its *exsA* transposon mutant (*exsA*::Tn) were used as positive and negative controls, respectively. (B) Immunoblotting detection of *exsCEBA* promoter-directed β-galactosidase. Total proteins were prepared from strain PAO1pClacZ (pcZ) and its derivatives. The expression levels were assayed by immunoblotting using antibodies against β-galactosidase. (C) Expression of P*exsC–lacZ* in *crc* deletion mutant under different growth conditions as indicated. Bacterial cultures were grown in Luria–Bertani (LB) medium, LB with 5.0 mmol/L nitrilotiracetic acid (LBnta), or Mins minimal medium. The data were the means of three replicates and error bar represents standard deviations.

To identify the genes that regulate the expression of T3SS in *P. aeruginosa*, the promoter of *exsCEBA* operon was fused to the reporter gene *lacZ* and the construct was integrated into the chromosome of the strain PAO1 to generate the reporter strain PAO1pClacZ as described previously (Zhou et al. 2007). A transposon mutant library of strain PAO1pClacZ was generated by random insertional mutagenesis using Mariner transposon, and the library was screened for genes implicated in the regulation of T3SS in *P. aeruginosa* under T3SS-inducing condition (by addition of 5 mmol/L calcium-depleted chelating regent – NTA to LB medium). One mutant, p39, was found to have dramatically reduced *exsCEBA* promoter-directed β-galactosidase activity (Fig. 1A). PCR and DNA sequencing analysis revealed that p39 contains a transposon insertion in the coding region of *crc* gene. To confirm the role of Crc in the modulation of *exsCEBA* expression, the wild-type *crc* gene was amplified by PCR from the genomic DNA of *P. aeruginosa* PAO1 and the fragment was cloned into a multicopy vector pUCP19 under the control of the *lac* promoter. The construct was introduced into the mutant p39 and the β-galactosidase activity assay showed that expression of *exsCEBA* was restored in the complemented strain (Fig. 1A). For further validation, we generated the *crc* in-frame deletion mutant using *P. aeruginosa* PAO1pClacZ as the parental strain. The assay results confirmed that deletion of *crc* reduced the expression of *exsCEBA* and *in trans* expression of the wild-type *crc* allele in Δ*crc* mutant restored the expression of *exsCEBA* to the level of its parental strain PAO1pClacZ (Fig. 1A, right). The β-galactosidase activity assay results were further supported by Western blot analysis on the abundance of the β-galactosidase protein (Fig. 1B). As Mins minimal medium was reported to be capable of maximizing the induction of T3SS (Nicas and Iglewski 1984), it is interesting to determine whether disruption of *crc* could still hamper *exsCEBA* expression in a strongly T3SS-inducing condition. The results showed the same pattern as observed in the LB + NTA medium (Fig. 1C). The findings indicate that Crc is critical for the expression of *exsCEBA* operon under various growth conditions. Taken together, the above results indicate that Crc positively regulates the expression of *exsCEBA*.

### Disruption of *crc* impaired the expression of *exoT*, *exsD*, and *popN* and attenuated T3SS-dependent cytotoxicity

**Figure 2:**
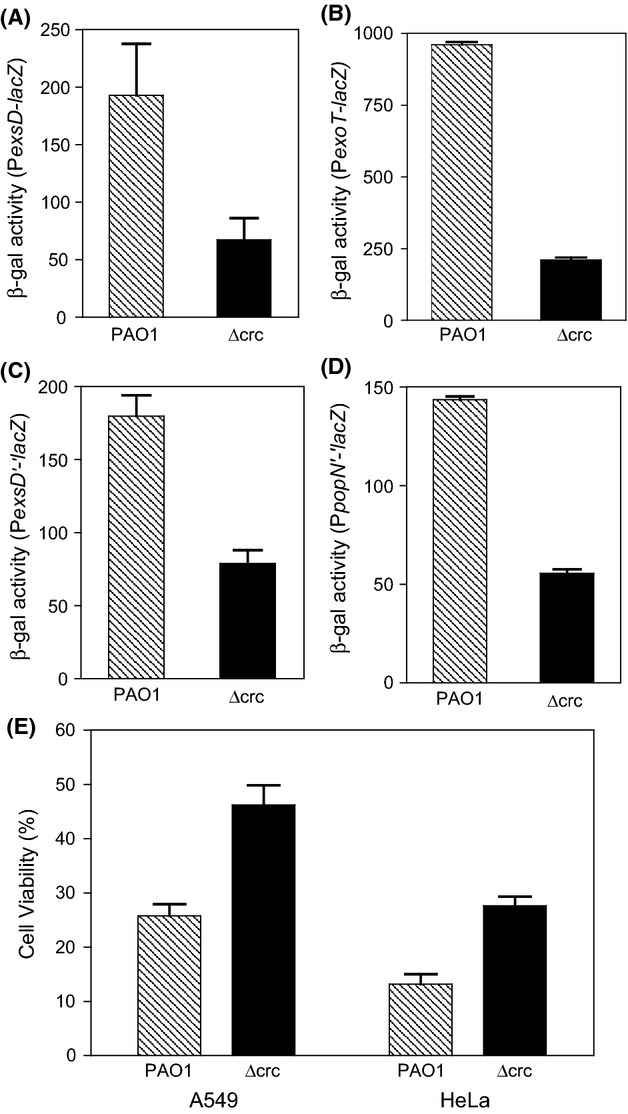
Reduced expression of *exoT*, *exsD*, and *popN* in *crc* deletion mutant. (A) and (B) Transcriptional expression of *exsD* (A) and *exoT* (B) in wild-type PAO1 and Δ*crc* mutant carrying a vector with P*exsD–lacZ* or P*exoT–lacZ* transcriptional reporter gene. (C) and (D) The translational expression of *exsD* (C) and *popN* (D) in wild-type PAO1 and Δ*crc* mutant carrying a vector with P*exsD’-‘lacZ* or P*popN’-‘lacZ* translational fusion reporter genes. Bacteria were grown at 37°C in Luria–Bertani medium containing 5.0 mmol/L nitrilotiracetic acid. (E) Reduced cytotoxicity in *crc* mutant in comparison with wild-type PAO1. Cytotoxicity was assayed using Cell Counting Kit-8 (Dojindo Molecular Technologies), which is based on the dehydrogenase activity detection in viable cells. HeLa and A549 cells were infected with bacterial cells at multiplicity of infection of 50. Absorbance at 450 nm was measured and the cell viability was calculated. The data were the means of four (A–D) or five (E) replicates and error bar represents standard deviations.

In addition to analyzing the expression of the master regulator *exsCEBA* operon in Δ*crc* mutant, we also tested the impact of Crc on expression of other T3SS genes. We first checked the transcription of *exsD* and *exoT* by measuring their promoter-directed β-galactosidase activity. *exsD* encodes an anti-activator of ExsA whose expression is positively regulated by ExsA (McCaw et al. 2002), and ExoT is a bifunctional type III cytotoxin exoenzyme T. The assay results showed that their expression was reduced drastically in the Δ*crc* mutant compared with the parental strain, either cultured in LB-NTA (Fig. 2A and B) or Mins medium (data not shown). Next, we tested the translational expression of *exsD* and *popN* by in-frame gene fusions to a truncated *lacZ* reporter gene (Heeb et al. 2000). As both proteins of ExsD and PopN are known for their negative regulatory role on T3SS, we wondered whether Crc exerts its influence on T3SS by inhibiting their translation. The β-galactosidase activity assay results showed that the translational expression of both genes was downregulated in the Δ*crc* mutant (Fig. 2C and D).

To test T3SS-dependent cytotoxicity toward mammalian cells, wild-type PAO1 and Δ*crc* mutant were used to infect the human epithelial cell lines Hela and A549. After 4-h infection, the cells were measured for dehydrogenase activity. As shown in Figure 2E, the cell viability in the sample infected with Δ*crc* strain was higher than that challenged with the wild-type PAO1, indicating that the Δ*crc* mutant was less cytotoxic. This result is agreeable with Linares's result that Crc reduces virulence in Dictyostelium (Linares et al. 2010).

### Crc modulates T3SS by influencing the expression level of ExsA

**Figure 3:**
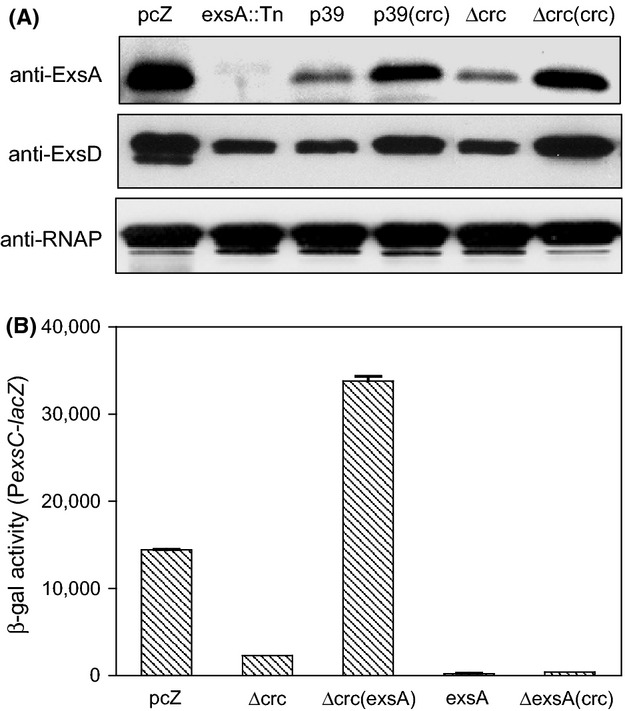
Influence of Crc on type III secretion system (T3SS) dependent on ExsA expression. (A) Immunoblotting detection of the expression level of ExsA and ExsD. Total proteins were prepared from strain PAO1pClacZ (pcZ) and its derivatives. The expression levels were assayed by immunoblotting using antibodies against ExsA, and ExsD as well as RNA polymerase, which serves as internal control. (B) *In trans* expression of *exsA* restored the T3SS gene expression in Δ*crc* mutant, but overexpression of *crc* in Δ*exsA* mutant failed to induce the expression of T3SS genes. In these *in trans* expression experiments, the empty vector pUCP19 was introduced into corresponding control strains for comparison analysis. Bacterial strains were grown under T3SS-inducing condition (Luria–Bertani + 5 mmol/L nitrilotiracetic acid).

As ExsA is a master regulator of T3SS, we tested whether the positive regulation of Crc on T3SS was exerted through ExsA. Immunoblotting using antibody against ExsA yielded much weaker signals from both of *crc* transposon and *crc* deletion mutants compared with the parental strain; whereas the *crc*-complemented strains showed stronger signals (Fig. 3A, anti-ExsA), indicating that the influence of Crc on T3SS was mediated by ExsA. Similarly, immunoblots of ExsD presented the same patterns (Fig. 3A, anti-ExsD), which correlates with previous observation that the expression of ExsD is under the control of the master regulator ExsA (McCaw et al. 2002).

To determine whether Crc regulates T3SS solely through ExsA, we tested the T3SS expression patterns by *in trans* expression of *exsA* in Δ*crc* mutant and by introducing wild-type *crc* into Δ*exsA* mutant, respectively. β-Galactosidase activity assay showed that overexpression of *exsA* in Δ*crc* mutant resulted in enhanced expression of *exsCEBA,* whereas overexpression of *crc* in Δ*exsA* had no effect on T3SS gene expression (Fig. 3B). Cumulatively, these results suggest that the influence of Crc on T3SS is through its direct or indirect regulatory role on the transcriptional expression of *exsA*.

### Effect of different carbon sources and Cbr/Crc signaling system on the regulation of T3SS

**Figure 4:**
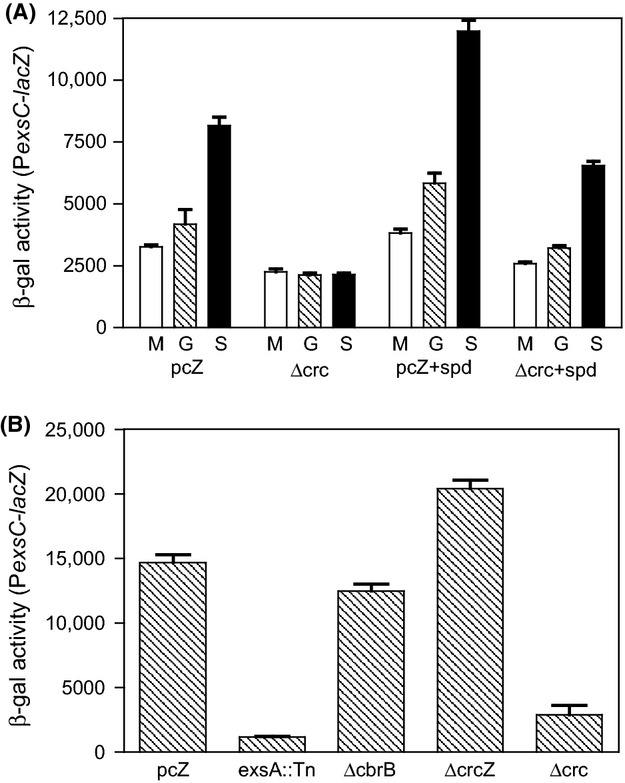
Effect of different carbon sources and Cbr/Crc signaling system on type III secretion system (T3SS) gene expression. (A) The transcriptional expression of P*exsC–lacZ* in response to different carbon sources with/without external addition of T3SS-inducer spermidine. Crc deletion mutant (Δ*crc*) and its parental strain PAO1pClacZ (pcZ) were grown in BSM minimal medium supplemented with either 40 mmol/L mannitol (M), 40 mmol/L glucose (G), or 40 mmol/L succinate (S) with/without 1 mmol/L spermidine (spd). (B) Effect of Cbr/Crc signaling system on the expression of *exsCEBA*. Bacterial strains were grown in Luria–Bertani medium with 5.0 mmol/L nitrilotiracetic acid at 37°C and their parental strain PAO1pclacZ (pcZ) and *exsA* mutant were used as positive and negative controls, respectively. The data were the means of three (A) or six (B) replicates and error bar represents standard deviations.

Crc and CbrA/CbrB two-component system have been demonstrated to play a role in catabolite repression and enabling *Pseudomonas* spp. to utilize various carbon sources (Nishijyo et al. 2001; Rojo 2010). To test whether different carbon sources could have a possible effect on T3SS in *P. aeruginosa*, three different carbon sources, that is, succinate, glucose, and mannitol were used and the expression of *exsCEBA* was measured. Among them, succinate is a preferred substrate, which can cause strong catabolite repression in *Pseudomonas*, glucose causes mild catabolite repression, whereas mannitol is unable to cause catabolite repression (Sonnleitner et al. 2009). These three carbon sources were each supplemented in BSM minimal medium, at a final concentration of 40 mmol/L with or without addition of 1 mmol/L spermidine, which is a known host signal that induces the transcription of T3SS genes in *P. aeruginosa* (Zhou et al. 2007). Δ*crc* mutant grew poorly in mannitol-supplemented medium, but addition of spermidine improved its growth (data not shown). Importantly, β-galactosidase activity assay of wild-type strain showed that maximal expression of *exsCEBA* was detected when succinate was used as carbon source, which seems to suggest that catabolite repression promotes T3SS expression. This notion was reinforced by the findings that glucose was better than mannitol in promoting the expression of *exsCEBA*. As expected, the influence of carbon source on T3SS expression was abolished when *crc* was deleted (Fig. 4A). Interestingly, we found that addition of spermidine partially restored the T3SS response to catabolite repression-dependent induction in the Δ*crc* mutant background (Fig. 4A). As the function of Crc is under the control of CbrA/CbrB two-component system and small RNA CrcZ, we next tested their effects on the expression of *exsCEBA*. Under T3SS-inducing conditions, the *cbrB* deletion mutant showed only a minor reduction in expression of *exsCEBA*, while the *crcZ* deletion mutant displayed an increased expression in comparison with their parental strain (Fig. 4B). These results indicate that the Cbr/Crc signaling system is involved in the regulation of T3SS in addition to its role in catabolite repression in *P. aeruginosa*.

### Transcriptome analysis revealed the mutation in *crc* downregulated the transcription of T3SS and T3SS-related genes

**Figure 5:**
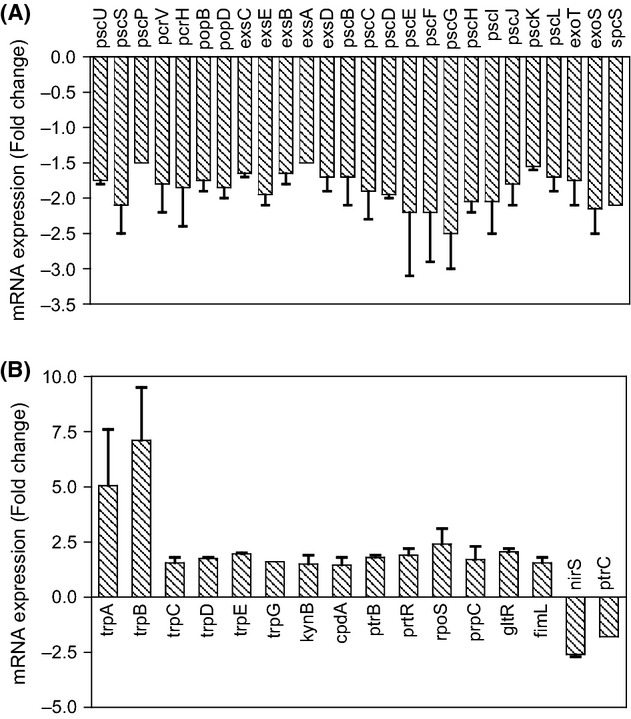
Expression of type III secretion system (T3SS) and T3SS-related genes in Δ*crc* mutant by transcriptome analysis. (A) Decreased transcripts of 26 T3SS genes in Δ*crc* mutant in comparison with its parental strain PAO1pClacZ. (B) Expression of T3SS-related genes in Δ*crc* mutant. Bacteria were cultured in Mins medium at 37°C and the cells were collected at OD_600_ ≍ 1.5. The data are shown as the transcriptional fold changes (Δ*crc* vs. PAO1pClacZ). The microarray was repeated twice and the mean data were presented.

To investigate the possible regulatory mechanisms of Crc on T3SS, global gene expression profiles of *crc* mutant and its parental strain PAO1pClacZ were examined by using the *P. aeruginosa* whole-genome microarray (Affymetrix) under calcium-depleted conditions. The results showed that a total of 425 genes were differentially regulated by at least twofold in mRNA expression by Crc. Among them, 208 genes were downregulated (Table S1) and 217 were upregulated in the Δ*crc* mutant (Table S2). Agreeable with the genetic analysis data, the transcriptional expression levels of 26 T3SS genes, including *exsCEBA*, *exsDpscBCDEFGHIJK*, *pcrVHpopBD*, *exoT,* and *exoS* etc., were decreased more than 1.5-fold in the Δ*crc* mutant (Fig. 5A). RT-PCR analysis confirmed the reduced expression of *exsC*, *exsD*, *pscF*, *popB,* and *exoT* in Δ*crc* mutant (Fig. S1).

**Figure 6:**
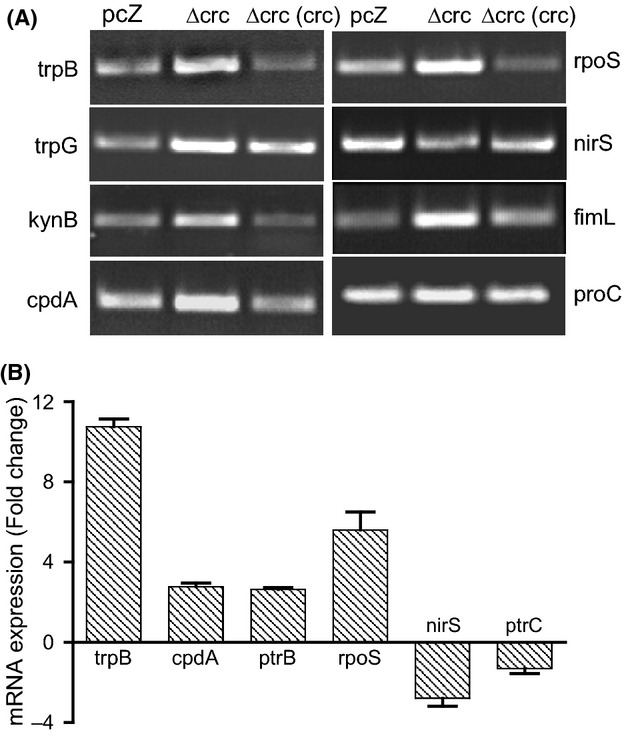
Transcriptional analysis by using real time polymerase chain reaction (RT-PCR) and real-time quantitative polymerase chain reaction (RT-qPCR) for selected type III secretion system (T3SS)-related genes. (A) RT-PCR analysis of the selected T3SS-related genes in Δ*crc* mutant, *crc*-complemented strain Δ*crc*(*crc*) and parental strain PAO1pclacZ (pcZ). The gene *proC* was used as internal control. (B) RT-qPCR analysis of several selected genes on their relative expression in Δ*crc* mutant in comparison with its parental strain PAO1pclacZ. Their expression levels were normalized against the reference gene *proC* and the data presented were the average of three experiments.

In addition, several genes associated with the regulation of T3SS (Diaz et al. 2011) were also found to be influenced by Crc (Fig. 5B). These genes include the *trp* operon *trp*ABCBEG, as well as *kynB*, *cpdA, ptrB, ptrR, prpC, rpoS, gltR, fimL, nirS,* and *ptrC* (see Discussion section for the description of these genes). Apart from *nirS* and *ptrC,* which were downregulated in *crc* mutant, other genes were upregulated in the mutant (≥1.5-fold change). Furthermore, *mgtE* and *aceE* were found also to be moderately upregulated by 1.4- and 1.3-fold, respectively. To confirm the expression patterns of these genes in Δ*crc* mutant and its parental strain, RT-PCR and real-time PCR were performed. The results confirmed that their expressions were influenced by Crc (Fig. 6).

## Discussion

Crc has been known to repress the expression of genes involved in assimilation of sugars, nitrogenated compounds, aromatic compounds, and hydrocarbons and in the transport of catabolites when preferred substrates, such as succinate, are present (Wolff et al. 1991; MacGregor et al. 1996; Hester et al. 2000). Here we showed that Crc positively regulated the expression of many T3SS genes in *P. aeruginosa* under T3SS-inducing condition. Detailed genetic and transcriptomic analyses indicate that Crc may exert a multifaceted effect on T3SS through various pathways to respond to different environmental signals. Our results provide further information to establish the links and connections between Cbr/Crc catabolite repression signaling system and T3SS regulatory network. It is possible that relief of catabolite repression by inactivation of Crc might have changed the intracellular level of some catabolites and such changes may repress the expression of T3SS genes. In fact, some catabolic pathways, such as histidine utilization, were reported to be severely depressed by catabolite repression when succinate was added in the medium (Potts and Clarke 1976), and the reduced level of histidine due to overexpression of genes involved in histidine transport and metabolism prevents expression of T3SS genes (Rietsch et al. 2004). In this study, we found that the tryptophan catabolic pathway is depressed by Crc-mediated catabolite repression (Figs. 5B, 6) and tryptophan metabolites, such as indole-3-acetic acid, kynurenine and PQS were reported to inhibit T3SS expression (Shen et al. 2008; Singh et al. 2010). These findings strongly suggest that catabolite repression may be implicated in modulation of T3SS in *P. aeruginosa*.

Crc is an RNA-binding protein, which was known to bind to an A-rich region located close to the ribosomal-binding site of target mRNAs to inhibit translation initiation (Moreno et al. 2007, 2009, 2010; Sonnleitner et al. 2009). On the basis of the above information and combining a previous bioinformatic study, which predicted 215 candidate genes containing A-rich motifs (AANAANAA) on their 5′ end of mRNA (Browne et al. 2010), we tried to locate key T3SS genes with the A-rich motif within or close to their ribosomal binding site. However, we could not find any. It seemed unlikely that the regulation of Crc on T3SS is through direct binding to the ribosomal-binding site of the key T3SS genes. However, when the same search was conducted on the Crc regulon, 56 genes containing the A-rich motif in their promoter region were identified (Table S3), but none of them belongs to T3SS regulon. These data may provide further information of the mechanism of regulation of other genes by Crc in *P. aeruginosa*.

Although Crc is known to inhibit target mRNA translation, only a few targets have been experimentally demonstrated, and these include *benR*, *alkS*, *xylR,* and *xylB* from *P. putida* and *amiE* from *P. aeruginosa* (Moreno et al. 2007, 2009, 2010; Sonnleitner et al. 2009). Our transcriptome profiles indicated that Crc indeed serves as a global regulator in controlling the expression of many genes, including that of the type III secretion system. Crc may influence on gene expression indirectly, but we still could not rigorously eliminate the possibility that Crc may also have a direct role in transcriptional regulation through its influence on mRNA stability. The fact that the *crc* regulon makes up to nearly 10% of the genome suggests that the mode of regulation by Crc is pleiotropic rather than specific. Comparing our transcriptomic data with Linares's proteomic data (both with fold change threshold set at 1.5), we found only 13 genes from our transcriptomic data in common with that of the proteomics data, and the latter has reported 66 differentially expressed proteins in the *crc* mutant (Linares et al. 2010). The same trend was observed in *P. putida*, where only an overlap of 23 genes were identified to be differentially transcribed in the transcriptomic assay of the total of 64 proteins controlled by Crc as shown by proteomics methods (Moreno et al. 2009). The difference between transcriptomic and proteomic analysis could reflect the different culture conditions, experimental errors, and/or posttranscriptional regulation, and the inherent disparities between protein and mRNA molecular stabilities. Our microarray data may provide the basic information for further exploring the different mechanisms of action for Crc.

In addition to the key regulatory genes of T3SS, several other genes, including *trp, kyn, cpdA, ptrB, ptrR, prpC, rpoS, gltR, fimL, nirS,* and *ptrC,* have been documented to play a role in the modulation of T3SS (Diaz et al. 2011), but their molecular regulatory mechanisms are largely unknown. Our microarray result suggested that in addition to the 26 T3SS genes with much reduced expression in *crc* mutant (Fig. 5A; Table S1), 16 genes known to be involved in the modulation of T3SS were also affected by Crc (Figs. 5B, 6; Tables S1, S2). Among these genes, seven genes (six *trp* genes and *kynB*) which are involved in tryptophan synthesis and catabolism were upregulated in *crc* mutant, in particular *trpAB* whose expression was increased by five- and sevenfold, respectively, in comparison with the parental strain (Fig. 5B; Table S2). Inactivation of either *trpA* or *kynA* gene resulted in a decrease in the T3SS-inhibiting activity, and further experiments found that the tryptophan derivatives, indole-3-acetic acid and 3-hydroxykynurenine, contribute to the inhibitory activity on T3SS expression (Shen et al. 2008). PQS, another tryptophan-derived quorum-sensing signaling molecule, was recently reported to cause inhibition of T3SS at elevated concentrations (Singh et al. 2010). We observed that deletion of *trpAB* in the *crc* mutant partially rescued the T3SS-deficient phenotype of Δ*crc* and deletion of *trpAB* in PAO1pClacZ also increased *exsCEBA* expression (Fig. S2A). Furthermore, the Δ*crc* mutant also showed increased PQS production (Fig. S2B). Thus, Crc might have modulated the T3SS in *P. aeruginosa* through inhibition of the tryptophan synthesis and catabolism pathway.

In addition, *ptrB*, *cpdA*, *rpoS,* and *prpC*, which were documented to possess negative regulatory effects on the expression of T3SS genes, were upregulated in the *crc* mutant (Figs. 5B, 6). *ptrB* encodes a small protein, which functions as a repressor of the expression of T3SS genes and the expression of *ptrB* is specifically repressed by the transcriptional regulator PrtR (Wu and Jin 2005). The expression of *prtR* was also upregulated in the *crc* mutant (Fig. 5B). *cpdA* encodes a cAMP phosphodiesterase, which hydrolyzes cAMP and decreases the intracellular cAMP concentration in *P. aeruginosa* which, in turn, reduces cAMP-Vfr-dependent virulence factor production, including T3SS (Fuchs et al. 2010). Inactivation of the stationary-phase sigma factor *rpoS* gene was found to be able to increase the expression of T3SS genes (Hogardt et al. 2004). Mutation in the citrate synthetase gene *prpC* resulted in an enhanced induction of T3SS (Rietsch and Mekalanos 2006). Notably, one gene, *nirS*, which encodes a nitrite reductase, was downregulated in the *crc* mutant (Fig. 5B). *nirS* was reported to have a positive effect on T3SS as its deletion mutant was defective in the expression of T3SS components in *P*. *aeruginosa* PA14 (van Alst et al. 2009). It is possible that the positive influence of Crc on the expression of T3SS genes may come from its global effect on the expression of these genes. However, regulation of Crc on other three T3SS-related genes, *gltR*, *fimL,* and *ptrC* seems inconsistent with its positive role in the modulation of T3SS. The *gltR* gene encodes a regulator for glucose transporter and was reported to have a positive effect on the expression of T3SS (Wolfgang et al. 2003). *fimL* encodes a protein required for type IV pili biogenesis and was also found to be required for T3SS-mediated cytotoxicity, and such activity is likely to occur, at least in part, via modulation of *vfr* expression and cAMP production (Whitchurch et al. 2005). *ptrC* gene encodes a small peptide and functions as T3SS repressor (Jin et al. 2011). Both *gltR* and *fimL* were found to be upregulated in *crc* mutant, but *ptrC* was shown to be downregulated in *crc* mutant (Fig. 5B). In addition, our microarray result also showed that among the 425 Crc-regulated genes, at least 39 of them encode proteins involved in transport (Figs. S1 and S2) and it is possible that some of them may have an influence on T3SS.

**Figure 7:**
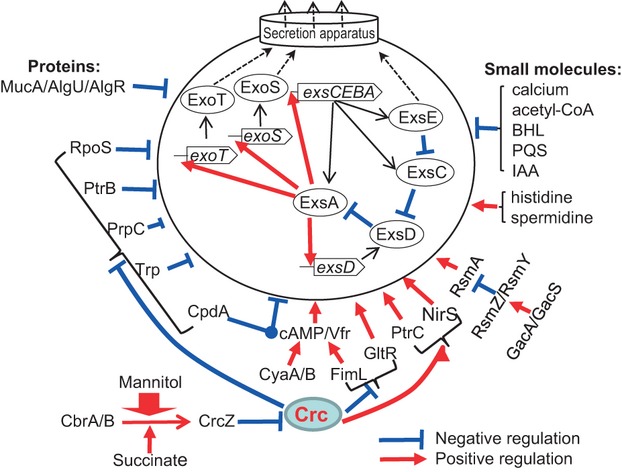
A working model of Crc in modulation of type III secretion system (T3SS) in *Pseudomonas aeruginosa*. Under the control of CrcZ and CbrAB two-component system, Crc positively influences the ExsA-dependent T3SS through modulating the expression of many genes and the production of some catabolites, which collectively contribute to regulation of T3SS. The key T3SS genes and encoding proteins are shown inside of the circle. The proteins and small molecules with documented regulatory role on T3SS are shown at outside the circle. Dashed line arrows indicate translocation, and solid arrows indicate positive regulation and the T bars indicate negative regulation. BHL, *N*-butanoyl-l-homoserine lactone; PQS, Pseudomonas quinolone signal; IAA, indole-3-acetic acid.

In summary, our data from detailed genetic and transcriptomic analysis confirmed the critical role of Crc in the regulation of T3SS in *P. aeruginosa*. The effect of Crc on T3SS was exerted through ExsA and was also linked to the Cbr/Crc signaling pathway. Furthermore, Crc seemed to modulate T3SS by participating in many known T3SS-related regulatory mechanisms. This complex and multifaceted effect of Crc may serve to fine-tune the T3SS to accurately sense and respond to environmental signals and nutrient sources, thereby creating a powerful virulence machinery (Fig. 7). Our results provide further information to establish the links and connections between catabolite repression and the complicated T3SS-regulatory network, which are essential for understanding the sophisticated regulatory mechanisms of the pathogen in infection and adaptation.

## Supporting Information

**Figure S1.** RT-PCR analysis of the selected T3SS genes in Δ*crc* mutant and parental strain PAO1pclacZ (pcZ).

**Figure S2.** Effect of *trpAB* deletion in Δ*crc* mutant (Δ*crc*/Δ*trpAB*) and parental strain PAO1pClacZ on *exsCEBA* expression (A) and PQS production (B).

**Table S1.** Downregulated genes in *crc* mutant under T3SS-induced condition (≤twofold).

**Table S2.** Up-regulated genes in *crc* mutant under T3SS-induced condition (≥twofold).

**Table S3.** Comparison of the *crc*-regulated genes and predicated *crc*-binding genes.
