# Supplementary figures and images for: The global regulator Crc plays a multifaceted role in modulation of type III secretion system in Pseudomonas aeruginosa
Source: Microbiologyopen. 2013 Jan 4;2(1):161–72. doi: 10.1002/mbo3.54 (PMC3584221; doi:10.1002/mbo3.54)

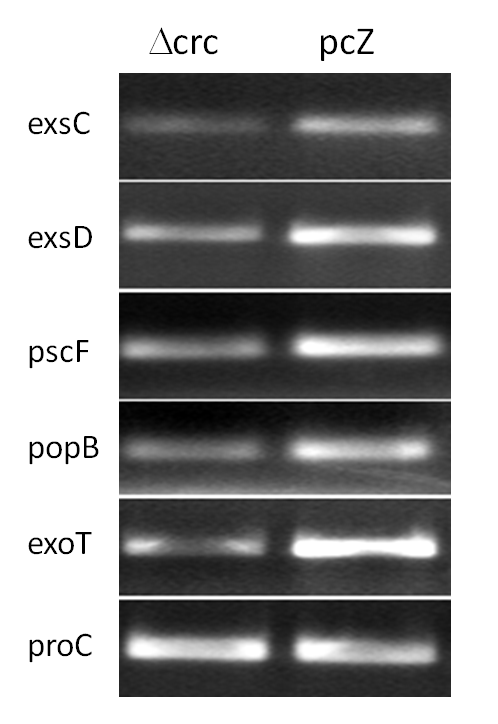

Supplement: Supplementary file 1 [file mbo30002-0161-SD1.tif]

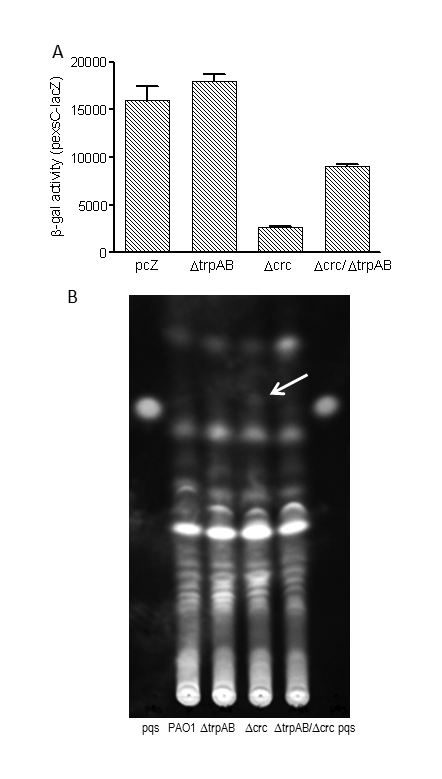

Supplement: Supplementary file 2 [file mbo30002-0161-SD2.tif]
