# Supplementary material for: The global regulator Crc plays a multifaceted role in modulation of type III secretion system in Pseudomonas aeruginosa
Source: Microbiologyopen. 2013 Jan 4;2(1):161–72. doi: 10.1002/mbo3.54 (PMC3584221; doi:10.1002/mbo3.54)
Supplement: Supplementary file 3 [file mbo30002-0161-SD3.pdf]

Table S1. Down-regulated genes in Δcrc mutant (≤2.0 fold)

PA#	Gene	Fold chang	SD	Protein
PA0045		-2.5	0.3	hypothetical protein
PA0046		-2.0	0.1	hypothetical protein
PA0109		-2.9	1.7	hypothetical protein
PA0179		-2.5	0.2	probable two-component response regulator
PA0269		-2.5	0.5	conserved hypothetical protein
PA0271		-2.3	1.1	hypothetical protein
PA0310		-2.3	1.0	hypothetical protein
PA0315		-2.2	0.6	hypothetical protein
PA0471		-4.5	3.6	probable transmembrane sensor
PA0472		-5.7	3.8	probable sigma-70 factor, ECF subfamily
PA0484		-2.7	0.8	conserved hypothetical protein
PA0505		-2.6	0.1	hypothetical protein
PA0509	nirN	-5.5	2.3	probable c-type cytochrome
PA0510		-5.3	1.8	probable uroporphyrin-III c-methyltransferase
PA0511	nirJ	-6.3	1.3	heme d1 biosynthesis protein NirJ
PA0512		-4.5	1.7	conserved hypothetical protein
PA0513		-5.1	0.5	probable transcriptional regulator
PA0514	nirL	-4.9	1.3	heme d1 biosynthesis protein NirL
PA0515		-4.6	0.6	probable transcriptional regulator
PA0516	nirF	-5.1	0.1	heme d1 biosynthesis protein NirF
PA0517	nirC	-4.1	0.1	probable c-type cytochrome precursor
PA0518	nirM	-4.1	1.1	cytochrome c-551 precursor
PA0519	nirS	-2.6	0.1	nitrite reductase precursor
PA0520	nirQ	-7.5	2.2	regulatory protein NirQ
PA0521		-15.9	2.1	probable cytochrome c oxidase subunit
PA0522		-8.4	3.4	hypothetical protein
PA0523	norC	-9.8	0.2	nitric-oxide reductase subunit C
PA0524	norB	-22.1	3.0	nitric-oxide reductase subunit B
PA0525		-37.3	24.0	probable dinitrification protein NorD
PA0558		-2.1	0.5	conserved hypothetical protein
PA0565		-2.3	0.0	conserved hypothetical protein
PA0586		-2.6	0.8	conserved hypothetical protein
PA0588		-2.0	0.4	conserved hypothetical protein
PA0589		-2.2	0.4	conserved hypothetical protein
PA0665		-2.2	0.0	conserved hypothetical protein
PA0672	hemO	-16.5	16.0	heme oxygenase
PA0713		-2.5	0.7	hypothetical protein
PA0743		-2.0	0.6	probable 3-hydroxyisobutyrate dehydrogenase
PA0840		-2.4	1.1	probable oxidoreductase
PA0872	phhA	-2.3	0.6	phenylalanine-4-hydroxylase
PA0918		-4.1	0.9	cytochrome b561
PA0929		-2.8	1.3	two-component response regulator
PA1081	flgF	-2.0	0.7	flagellar basal-body rod protein FlgF
PA1117		-2.1	0.3	hypothetical protein
PA1172	napC	-4.5	2.6	cytochrome c-type protein NapC
PA1173	napB	-4.1	1.3	cytochrome c-type protein NapB precursor
PA1174	napA	-3.1	0.6	periplasmic nitrate reductase protein NapA
PA1175	napD	-2.0	0.3	NapD protein of periplasmic nitrate reductase

PA1176	napF	-2.2	0.4	ferredoxin protein NapF
PA1177	napE	-2.8	0.8	periplasmic nitrate reductase protein NapE
PA1183	dctA	-2.1	0.8	C4-dicarboxylate transport protein
PA1287		-2.3	1.1	probable glutathione peroxidase
PA1300		-5.2	4.2	probable sigma-70 factor, ECF subfamily
PA1305		-2.0	0.4	hypothetical protein
PA1314		-6.4	5.9	hypothetical protein
PA1317	cyoA	-3.2	1.6	cytochrome o ubiquinol oxidase subunit II
PA1318	cyoB	-2.3	0.3	cytochrome o ubiquinol oxidase subunit I
PA1333		-2.3	0.4	hypothetical protein
PA1366		-2.0	0.4	hypothetical protein
PA1404		-2.3	0.5	hypothetical protein
PA1473		-2.1	0.1	hypothetical protein
PA1677		-2.0	0.1	conserved hypothetical protein
PA1679		-2.4	0.9	hypothetical protein
PA1692		-2.1	0.6	probable translocation protein in type III secretion
PA1711	exsE	-2.0	0.2	ExsE
PA1717	pscD	-2.0	0.1	type III export protein PscD
PA1719	pscF	-2.2	1.0	type III export protein PscF
PA1720	pscG	-2.5	0.7	type III export protein PscG
PA1721	pscH	-2.1	0.2	type III export protein PscH
PA1722	pscI	-2.1	0.6	type III export protein PscI
PA1728		-2.5	1.4	hypothetical protein
PA1732		-2.0	0.7	conserved hypothetical protein
PA1733		-2.0	0.2	conserved hypothetical protein
PA1745		-2.1	0.2	hypothetical protein
PA1774	cfrX	-2.0	0.4	CfrX protein
PA1847		-5.0	0.9	conserved hypothetical protein
PA1870		-2.4	1.0	hypothetical protein
PA1912	femI	-3.2	1.9	ECF sigma factor, FemI
PA1964		-2.5	0.9	probable ATP-binding component of ABC transporter
PA2024		-6.5	2.5	probable ring-cleaving dioxygenase
PA2121		-2.0	0.6	probable transcriptional regulator
PA2146		-2.8	0.2	conserved hypothetical protein
PA2190		-2.1	0.4	conserved hypothetical protein
PA2321		-3.8	1.5	gluconokinase
PA2322		-3.4	2.0	gluconate permease
PA2375		-3.2	1.8	hypothetical protein
PA2398	fpvA	-5.1	3.1	ferripyoverdine receptor
PA2404		-2.1	0.8	hypothetical protein
PA2405		-2.0	0.6	hypothetical protein
PA2481		-2.4	1.2	hypothetical protein
PA2482		-2.9	0.7	probable cytochrome c
PA2483		-3.7	1.3	conserved hypothetical protein
PA2532	tpx	-2.5	0.6	thiol peroxidase
PA2550		-4.7	3.5	probable acyl-CoA dehydrogenase
PA2666		-2.1	0.3	probable 6-pyruvoyl tetrahydrobiopterin synthase
PA2686	pfeR	-2.2	1.0	two-component response regulator PfeR
PA2691		-11.1	9.6	conserved hypothetical protein
PA2692		-2.6	0.8	probable transcriptional regulator

PA2746		-3.0	1.1	hypothetical protein
PA2761		-2.3	0.0	hypothetical protein
PA2788		-3.0	1.1	probable chemotaxis transducer
PA2790		-2.7	1.7	hypothetical protein
PA2840		-7.3	3.9	probable ATP-dependent RNA helicase
PA2897		-2.0	0.4	probable transcriptional regulator
PA2937		-2.6	0.3	hypothetical protein
PA3009		-3.1	0.8	hypothetical protein
PA3081		-2.2	0.7	conserved hypothetical protein
PA3205		-2.5	0.5	hypothetical protein
PA3266	capB	-2.5	0.8	cold acclimation protein B
PA3281		-3.6	2.5	hypothetical protein
PA3282		-4.5	3.8	hypothetical protein
PA3283		-6.4	4.2	conserved hypothetical protein
PA3284		-5.8	3.3	hypothetical protein
PA3307		-3.2	1.3	hypothetical protein
PA3391	nosR	-2.7	0.3	regulatory protein NosR
PA3392	nosZ	-8.0	1.2	nitrous-oxide reductase precursor
PA3393	nosD	-7.0	2.3	NosD protein
PA3394	nosF	-6.2	4.8	NosF protein
PA3395	nosY	-4.7	0.6	NosY protein
PA3396	nosL	-3.6	2.3	NosL protein
PA3415		-2.4	1.2	probable dihydrolipoamide acetyltransferase
PA3418	ldh	-3.9	1.4	leucine dehydrogenase
PA3436		-2.6	1.3	hypothetical protein
PA3530		-17.4	18.7	conserved hypothetical protein
PA3577		-2.5	0.9	hypothetical protein
PA3578		-2.0	0.6	conserved hypothetical protein
PA3688		-6.2	5.2	hypothetical protein
PA3723		-4.1	1.6	probable FMN oxidoreductase
PA3737	dsbC	-2.0	0.1	thiol:disulfide interchange protein DsbC
PA3808		-4.0	0.5	conserved hypothetical protein
PA3809	fdx2	-4.3	1.3	ferredoxin [2Fe-2S]
PA3810	hscA	-4.5	1.8	heat shock protein HscA
PA3811	hscB	-6.0	1.8	heat shock protein HscB
PA3812	iscA	-4.6	0.8	probable iron-binding protein IscA
PA3813	iscU	-5.3	0.1	probable iron-binding protein IscU
PA3814	iscS	-5.1	0.1	L-cysteine desulfurase (pyridoxal phosphate-dependent)
PA3815		-5.2	1.9	conserved hypothetical protein
PA3817		-2.1	0.4	probable methyltransferase
PA3818		-3.6	1.6	extragenic suppressor protein SuhB
PA3841	exoS	-2.2	0.5	exoenzyme S
PA3842		-2.1	0.0	probable chaperone
PA3843		-2.8	0.4	hypothetical protein
PA3846		-2.1	0.6	hypothetical protein
PA3880		-2.0	0.3	conserved hypothetical protein
PA3899		-6.3	5.3	probable sigma-70 factor, ECF subfamily
PA3900		-2.6	1.1	probable transmembrane sensor
PA3920		-23.5	22.7	probable metal transporting P-type ATPase
PA3928		-2.6	0.8	hypothetical protein

PA3929	cioB	-3.0	1.3	cyanide insensitive terminal oxidase
PA3930	cioA	-5.1	1.0	cyanide insensitive terminal oxidase
PA3967		-2.0	0.4	hypothetical protein
PA3986		-2.2	0.9	hypothetical protein
PA4090		-2.8	0.5	hypothetical protein
PA4155		-10.9	9.4	hypothetical protein
PA4156		-39.8	30.7	probable TonB-dependent receptor
PA4158	fepC	-2.7	1.2	ferric enterobactin transport protein FepC
PA4159	fepB	-3.3	1.3	ferrienterobactin-binding periplasmic protein precursor FepB
PA4218		-2.8	1.2	probable transporter
PA4219		-2.9	1.2	hypothetical protein
PA4220		-5.0	2.5	hypothetical protein
PA4221	fptA	-4.7	0.8	Fe(III)-pyochelin outer membrane receptor precursor
PA4228	pchD	-3.1	1.2	pyochelin biosynthesis protein PchD
PA4229	pchC	-2.9	0.9	pyochelin biosynthetic protein PchC
PA4230	pchB	-3.1	0.5	salicylate biosynthesis protein PchB
PA4231	pchA	-2.6	0.8	salicylate biosynthesis isochorismate synthase
PA4235	bfrA	-2.2	0.4	bacterioferritin
PA4468	sodM	-5.2	4.6	superoxide dismutase
PA4469		-13.4	9.8	hypothetical protein
PA4470	fumC1	-19.9	21.3	fumarate hydratase
PA4471		-20.8	20.5	hypothetical protein
PA4515		-4.4	0.8	conserved hypothetical protein
PA4516		-2.2	0.8	hypothetical protein
PA4525	pilA	-2.1	0.3	type 4 fimbrial precursor PilA
PA4550	fimU	-2.2	0.8	type 4 fimbrial biogenesis protein FimU
PA4551	pilV	-2.3	0.8	type 4 fimbrial biogenesis protein PilV
PA4552	pilW	-2.4	1.2	type 4 fimbrial biogenesis protein PilW
PA4555	pilY2	-3.0	0.6	type 4 fimbrial biogenesis protein PilY2
PA4556	pilE	-2.8	0.3	type 4 fimbrial biogenesis protein PilE
PA4570		-7.2	5.7	hypothetical protein
PA4605		-2.1	0.3	conserved hypothetical protein
PA4607		-2.0	0.1	hypothetical protein
PA4610		-4.5	1.3	hypothetical protein
PA4615		-5.7	1.8	probable oxidoreductase
PA4619		-3.0	1.6	probable c-type cytochrome
PA4620		-5.0	1.6	hypothetical protein
PA4621		-2.3	1.1	probable oxidoreductase
PA4656		-2.7	1.2	conserved hypothetical protein
PA4657		-2.0	0.4	hypothetical protein
PA4658		-6.3	2.1	hypothetical protein
PA4659		-5.8	4.6	probable transcriptional regulator
PA4660	phr	-4.1	2.1	deoxyribodipyrimidine photolyase
PA4683		-4.2	0.8	hypothetical protein
PA4692		-2.5	1.1	conserved hypothetical protein
PA4702		-2.6	0.9	hypothetical protein
PA4709		-3.2	1.1	probable hemin degrading factor
PA4746		-3.2	1.5	conserved hypothetical protein
PA4753		-2.1	0.4	conserved hypothetical protein
PA4878		-2.9	1.0	probable transcriptional regulator

PA4896		-6.6	5.1	probable sigma-70 factor, ECF subfamily
PA5115		-2.8	1.2	conserved hypothetical protein
PA5119	glnA	-2.2	0.2	glutamine synthetase
PA5217		-2.4	1.2	probable binding protein component of ABC iron transporter
PA5275		-3.5	2.3	conserved hypothetical protein
PA5289		-2.0	0.6	hypothetical protein
PA5408		-2.4	0.2	hypothetical protein
PA5409		-2.5	1.1	hypothetical protein
PA5438		-2.0	0.6	probable transcriptional regulator
PA5531	tonB	-3.6	1.6	TonB protein
