# Supplementary material for: The global regulator Crc plays a multifaceted role in modulation of type III secretion system in Pseudomonas aeruginosa
Source: Microbiologyopen. 2013 Jan 4;2(1):161–72. doi: 10.1002/mbo3.54 (PMC3584221; doi:10.1002/mbo3.54)
Supplement: Supplementary file 5 [file mbo30002-0161-SD4.pdf]

Table S2. Up-regulated genes in Δcrc mutant (≥2.0 fold)

PA#	Gene	Fold change	SD	Protein
PA0035	trpA	5.1	3.6	tryptophan synthase alpha chain
PA0036	trpB	7.1	3.4	tryptophan synthase beta chain
PA0117		2.1	0.8	probable short chain dehydrogenase
PA0155	pcaR	2.4	0.4	transcriptional regulator PcaR
PA0167		3.0	2.0	probable transcriptional regulator
PA0191		3.0	1.8	probable transcriptional regulator
PA0195	pntAA	2.0	0.6	putative NAD(P) transhydrogenase, subunit alpha part 1
PA0201		2.5	0.7	hypothetical protein
PA0281	cysW	5.4	5.3	sulfate transport protein CysW
PA0282	cysT	4.1	2.9	sulfate transport protein CysT
PA0283	sbp	7.4	6.9	sulfate-binding protein precursor
PA0284		6.4	5.6	hypothetical protein
PA0291	oprE	2.2	0.6	Anaerobically-induced outer membrane porin OprE precursor
PA0302	spuF	2.0	0.5	polyamine transport protein PotG
PA0336	ygdP	2.5	1.3	Nudix hydrolase YgdP
PA0433		3.2	0.1	hypothetical protein
PA0434		2.9	1.1	hypothetical protein
PA0527	dnr	6.0	3.5	transcriptional regulator Dnr
PA0545		3.5	2.5	hypothetical protein
PA0546	metK	2.7	0.8	methionine adenosyltransferase
PA0548	tktA	2.1	0.6	transketolase
PA0552	pgk	2.1	0.5	phosphoglycerate kinase
PA0577	dnaG	2.3	0.9	DNA primase
PA0609	trpE	2.0	0.1	anthranilate synthetase component I
PA0781		2.5	1.2	hypothetical protein
PA0841		2.2	0.6	hypothetical protein
PA1072	braE	3.0	1.3	branched-chain amino acid transport protein BraE
PA1073	braD	4.6	2.8	branched-chain amino acid transport protein BraD
PA1074	braC	2.1	0.2	branched-chain amino acid transport protein BraC
PA1076		2.1	0.4	hypothetical protein
PA1126		2.3	0.1	hypothetical protein
PA1127		2.7	0.0	probable oxidoreductase
PA1150	pys2	2.1	0.4	pyocin S2
PA1156	nrdA	2.7	1.1	NrdA, catalytic component of class Ia ribonucleotide reductase
PA1159		4.5	3.9	probable cold-shock protein
PA1196		2.4	1.1	probable transcriptional regulator
PA1197		2.0	0.7	hypothetical protein
PA1198		2.4	0.4	conserved hypothetical protein
PA1228		2.4	0.1	hypothetical protein
PA1263		2.6	1.4	hypothetical protein
PA1288		2.4	0.9	probable outer membrane protein precursor
PA1293		2.2	0.5	hypothetical protein
PA1556		9.8	11.7	probable cytochrome c oxidase subunit
PA1557		15.5	19.7	probable cytochrome oxidase subunit (cbb3-type)
PA1596	htpG	3.9	2.4	heat shock protein HtpG
PA1673		3.1	1.7	hypothetical protein
PA1756	cysH	2.2	0.4	3'-phosphoadenosine-5'-phosphosulfate reductase
PA1789		5.7	5.3	hypothetical protein

PA1838	cysI	3.7	2.8	sulfite reductase
PA1922		2.0	0.3	probable TonB-dependent receptor
PA1927	metE	2.4	0.6	5-methyltetrahydropteroyltriglutamate-homocysteine S-methyltransferase
PA1984		2.1	0.3	probable aldehyde dehydrogenase
PA2009	hmgA	2.1	0.8	homogentisate 1,2-dioxygenase
PA2013	liuC	2.0	0.3	putative 3-methylglutaconyl-CoA hydratase
PA2015	liuA	4.9	3.6	putative isovaleryl-CoA dehydrogenase
PA2016	liuR	2.8	1.6	regulator of liu genes
PA2027		4.5	1.8	hypothetical protein
PA2126		2.0	0.7	conserved hypothetical protein
PA2127		3.9	1.5	conserved hypothetical protein
PA2204		9.3	8.8	probable binding protein component of ABC transporter
PA2310		2.5	0.7	hypothetical protein
PA2312		3.0	1.8	probable transcriptional regulator
PA2338		7.6	0.6	probable binding protein component of ABC maltose/mannitol transporter
PA2339		6.8	4.6	probable binding-protein-dependent maltose/mannitol transport protein
PA2340		7.8	5.8	probable binding-protein-dependent maltose/mannitol transport protein
PA2341		3.8	1.3	probable ATP-binding component of ABC maltose/mannitol transporter
PA2342	mtlD	3.8	1.4	mannitol dehydrogenase
PA2343	mtlY	3.5	0.7	xylulose kinase
PA2344	mtlZ	2.2	0.4	fructokinase
PA2359		5.0	4.0	probable transcriptional regulator
PA2379		2.8	0.4	probable oxidoreductase
PA2533		2.7	0.4	probable sodium:alanine symporter
PA2594		2.0	0.4	conserved hypothetical protein
PA2747		2.1	0.2	hypothetical protein
PA2760		3.9	2.0	probable outer membrane protein precursor
PA2811		2.1	0.6	probable permease of ABC-2 transporter
PA2812		2.4	0.8	probable ATP-binding component of ABC transporter
PA2826		2.3	1.1	probable glutathione peroxidase
PA2849		2.9	1.6	probable transcriptional regulator
PA2886	atuA	2.1	0.7	expressed protein with apparent function in citronellol catabolism
PA2887	atuB	3.3	2.5	putative dehydrogenase involved in catabolism of citronellol
PA2888	atuC	3.0	1.6	geranyl-CoA carboxylase, beta-subunit
PA2913		2.1	0.6	hypothetical protein
PA3001		2.0	0.6	probable glyceraldehyde-3-phosphate dehydrogenase
PA3011	topA	2.0	0.4	DNA topoisomerase I
PA3027		2.0	0.2	probable transcriptional regulator
PA3118	leuB	2.6	1.2	3-isopropylmalate dehydrogenase
PA3119		2.3	0.8	conserved hypothetical protein
PA3120	leuD	3.6	2.0	3-isopropylmalate dehydratase small subunit
PA3121	leuC	4.9	2.4	3-isopropylmalate dehydratase large subunit
PA3126	ibpA	2.9	0.9	heat-shock protein IbpA
PA3161	himD	2.0	0.1	integration host factor beta subunit
PA3181		2.2	0.2	2-keto-3-deoxy-6-phosphogluconate aldolase
PA3182	pgl	2.0	0.2	6-phosphogluconolactonase
PA3183	zwf	2.2	0.3	glucose-6-phosphate 1-dehydrogenase
PA3191		2.1	0.3	probable two-component sensor
PA3192	gltR	2.1	0.2	two-component response regulator GltR
PA3195	gapA	2.1	0.4	glyceraldehyde 3-phosphate dehydrogenase

PA3215		2.1	0.4	probable transcriptional regulator
PA3243	minC	2.1	0.8	cell division inhibitor MinC
PA3278		3.1	2.3	hypothetical protein
PA3300	fadD2	2.6	1.2	long-chain-fatty-acid--CoA ligase
PA3309		2.6	1.2	conserved hypothetical protein
PA3314		2.5	1.1	probable ATP-binding component of ABC transporter
PA3337	rfaD	3.8	3.1	ADP-L-glycero-D-mannoheptose 6-epimerase
PA3446		4.0	2.3	conserved hypothetical protein
PA3450		5.6	4.7	probable antioxidant protein
PA3537	argF	2.7	0.4	ornithine carbamoyltransferase, anabolic
PA3572		2.2	1.0	hypothetical protein
PA3581	glpF	2.9	0.4	glycerol uptake facilitator protein
PA3582	glpK	2.7	0.1	glycerol kinase
PA3583	glpR	2.3	0.1	glycerol-3-phosphate regulon repressor
PA3613		3.3	2.2	hypothetical protein
PA3622	rpoS	2.4	1.0	sigma factor RpoS
PA3730		2.1	0.7	hypothetical protein
PA3731		2.5	1.1	conserved hypothetical protein
PA3732		2.8	0.4	conserved hypothetical protein
PA3785		2.7	0.8	conserved hypothetical protein
PA3820	secF	2.1	0.6	secretion protein SecF
PA3836		3.5	0.9	hypothetical protein
PA3837		3.1	2.1	probable permease of ABC transporter
PA3838		2.8	1.6	probable ATP-binding component of ABC transporter
PA3859		2.0	0.5	carboxylesterase
PA3887	nhaP	2.5	1.0	Na+/H+ antiporter NhaP
PA3925		4.1	2.1	probable acyl-CoA thiolase
PA3931		3.5	1.8	conserved hypothetical protein
PA3932		2.1	0.6	probable transcriptional regulator
PA3935	tauD	2.3	0.8	taurine dioxygenase
PA3936		2.7	1.1	probable permease of ABC taurine transporter
PA3937		4.5	2.4	probable ATP-binding component of ABC taurine transporter
PA3938		5.9	2.3	probable periplasmic taurine-binding protein precursor
PA3965		2.2	0.6	probable transcriptional regulator
PA4064		2.2	0.9	probable ATP-binding component of ABC transporter
PA4067	oprG	3.0	2.1	Outer membrane protein OprG precursor
PA4094		2.1	0.6	probable transcriptional regulator
PA4170		3.9	2.1	hypothetical protein
PA4192		4.3	3.5	probable ATP-binding component of ABC transporter
PA4193		2.4	0.7	probable permease of ABC transporter
PA4195		3.3	1.4	probable binding protein component of ABC transporter
PA4198		2.7	0.2	probable AMP-binding enzyme
PA4286		2.0	0.2	hypothetical protein
PA4307	pctC	3.9	1.7	chemotactic transducer PctC
PA4328		4.6	3.0	hypothetical protein
PA4348		8.6	8.2	conserved hypothetical protein
PA4358		2.8	1.5	probable ferrous iron transport protein
PA4428	sspA	2.4	1.1	stringent starvation protein A
PA4429		3.0	1.9	probable cytochrome c1 precursor
PA4430		3.7	2.8	probable cytochrome b

PA4431		2.7	1.2	probable iron-sulfur protein
PA4442	cysN	4.6	4.1	ATP sulfurylase GTP-binding subunit/APS kinase
PA4443	cysD	4.7	3.0	ATP sulfurylase small subunit
PA4447	hisC1	2.3	1.0	histidinol-phosphate aminotransferase
PA4448	hisD	2.1	0.6	histidinol dehydrogenase
PA4542	clpB	2.1	0.7	ClpB protein
PA4548		2.0	0.4	probable D-amino acid oxidase
PA4571		3.3	1.9	probable cytochrome c
PA4600	nfxB	2.0	0.6	transcriptional regulator NfxB
PA4632		2.5	0.8	hypothetical protein
PA4672		2.2	0.3	peptidyl-tRNA hydrolase
PA4675		3.2	0.6	probable TonB-dependent receptor
PA4729	panB	2.7	0.6	3-methyl-2-oxobutanoate hydroxymethyltransferase
PA4730	panC	2.8	0.5	pantoate--beta-alanine ligase
PA4762	grpE	2.2	1.0	heat shock protein GrpE
PA4773		2.0	0.4	hypothetical protein
PA4787		2.5	0.8	probable transcriptional regulator
PA4852		2.0	0.1	conserved hypothetical protein
PA4853	fis	2.1	0.3	DNA-binding protein Fis
PA4854	purH	2.3	1.0	phosphoribosylaminoimidazolecarboxamide formyltransferase
PA4863		2.3	0.9	hypothetical protein
PA4864	ureD	2.1	0.7	urease accessory protein
PA4866		2.3	0.8	conserved hypothetical protein
PA4917		2.5	0.5	hypothetical protein
PA4918		4.5	0.9	hypothetical protein
PA4919	pncB1	3.4	1.1	nicotinate phosphoribosyltransferase
PA4973	thiC	2.1	0.6	thiamin biosynthesis protein ThiC
PA5024		2.2	0.5	conserved hypothetical protein
PA5030		2.1	0.2	probable major facilitator superfamily (MFS) transporter
PA5075		2.1	0.5	probable permease of ABC transporter
PA5080		2.0	0.4	prolyl aminopeptidase
PA5131	pgm	2.2	0.1	phosphoglycerate mutase
PA5152		4.2	1.1	probable ATP-binding component of ABC transporter
PA5153		3.9	1.7	amino acid ABC transporter periplasmic binding protein
PA5154		3.5	1.5	probable permease of ABC transporter
PA5155		2.4	1.2	amino acid ABC transporter membrane protein
PA5167		5.3	1.6	probable c4-dicarboxylate-binding protein
PA5168		9.8	4.6	probable dicarboxylate transporter
PA5169		10.2	5.3	probable C4-dicarboxylate transporter
PA5170	arcD	5.0	2.3	arginine/ornithine antiporter
PA5171	arcA	2.5	0.1	arginine deiminase
PA5172	arcB	2.3	0.5	ornithine carbamoyltransferase, catabolic
PA5173	arcC	3.0	1.3	carbamate kinase
PA5208		2.2	0.6	conserved hypothetical protein
PA5214	gcvH1	2.0	0.6	glycine cleavage system protein H1
PA5231		4.8	4.3	probable ATP-binding/permease fusion ABC transporter
PA5232		3.9	2.3	conserved hypothetical protein
PA5301		2.5	0.8	probable transcriptional regulator
PA5324		3.8	1.3	probable transcriptional regulator
PA5347		2.1	0.3	hypothetical protein

PA5348		5.4	0.9	probable DNA-binding protein
PA5351	rubA1	3.8	2.3	Rubredoxin 1
PA5414		2.0	0.3	hypothetical protein
PA5427	adhA	2.3	0.6	alcohol dehydrogenase
PA5436		2.7	1.6	probable biotin carboxylase subunit of a transcarboxylase
PA5440		2.1	0.8	probable peptidase
PA5442		3.0	1.4	conserved hypothetical protein
PA5450	wzt	3.1	1.0	ABC subunit of A-band LPS efflux transporter
PA5454	rmd	2.7	1.4	oxidoreductase Rmd
PA5455		3.5	1.8	hypothetical protein
PA5456		3.0	1.6	hypothetical protein
PA5457		2.8	1.0	hypothetical protein
PA5458		2.6	0.8	hypothetical protein
PA5459		2.4	0.6	hypothetical protein
PA5475		3.9	3.1	hypothetical protein
PA5536		2.0	0.4	conserved hypothetical protein
PA5545		2.3	0.9	conserved hypothetical protein
PA5556	atpA	2.1	0.8	ATP synthase alpha chain
PA5557	atpH	2.2	0.6	ATP synthase delta chain
