# Supplementary material for: The global regulator Crc plays a multifaceted role in modulation of type III secretion system in Pseudomonas aeruginosa
Source: Microbiologyopen. 2013 Jan 4;2(1):161–72. doi: 10.1002/mbo3.54 (PMC3584221; doi:10.1002/mbo3.54)
Supplement: Supplementary file 7 [file mbo30002-0161-SD5.pdf]

Table S3 Compareson of the crc-regulated genes and predicated crc-binding genes

PA#	Gene	Fold change	SD	Protein
PA0117		**2.1**	**0.8**	probable short chain dehydrogenase
PA0129	gabP	**1.7**	**0.1**	gamma-aminobutyrate permease
PA0133		**2.3**	**1.4**	probable transcriptional regulator
PA0430	metF	**1.8**	**0.4**	5,10-methylenetetrahydrofolate reductase
PA0585		**-1.7**	**1.0**	hypothetical protein
PA0782	putA	**-1.5**	**0.1**	proline dehydrogenase PutA
PA0783	putP	**1.7**	**0.1**	sodium/proline symporter PutP
PA0831	oruR	**1.6**	**0.0**	transcriptional regulator OruR
PA0872	phhA	**-2.3**	**0.6**	phenylalanine-4-hydroxylase
PA1617		**2.0**	**0.8**	probable AMP-binding enzyme
PA1991		**1.6**	**0.2**	probable iron-containing alcohol dehydrogenase
PA2081	kynB	**1.5**	**0.6**	kynurenine formamidase, KynB
PA2332		**1.5**	**0.2**	probable transcriptional regulator
PA2338		**7.5**	**0.7**	probable binding protein component of ABC maltose/mannitol transporter
PA2482		**-2.9**	**0.7**	probable cytochrome c
PA2760		**3.9**	**2.0**	probable outer membrane protein precursor
PA2776		**1.6**	**0.0**	conserved hypothetical protein
PA2886	atuA	**2.1**	**0.7**	expressed protein with apparent function in citronellol catabolism
PA2890	atuE	**1.8**	**0.0**	putative isohexenylglutaconyl-CoA hydratase
PA3027		**2.0**	**0.2**	probable transcriptional regulator
PA3038		**1.7**	**0.5**	probable porin
PA3161	himD	**2.0**	**0.1**	integration host factor beta subunit
PA3183	zwf	**2.2**	**0.2**	glucose-6-phosphate 1-dehydrogenase
PA3190		**1.6**	**0.5**	probable binding protein component of ABC sugar transporter
PA3215		**2.1**	**0.4**	probable transcriptional regulator
PA3283		**-6.4**	**4.3**	conserved hypothetical protein
PA3300	fadD2	**2.5**	**1.2**	long-chain-fatty-acid--CoA ligase
PA3482	metG	**1.7**	**0.5**	methionyl-tRNA synthetase
PA3537	argF	**2.6**	**0.3**	ornithine carbamoyltransferase, anabolic
PA3959		**1.6**	**0.7**	hypothetical protein
PA4070		**1.7**	**0.6**	probable transcriptional regulator
PA4090		**-2.8**	**0.5**	hypothetical protein
PA4094		**2.1**	**0.6**	probable transcriptional regulator
PA4247	rplR	**1.5**	**0.2**	50S ribosomal protein L18
PA4471		**-20.8**	**20.5**	hypothetical protein
PA4570		**-7.1**	**5.7**	hypothetical protein
PA4571		**3.2**	**1.9**	probable cytochrome c
PA4733	acsB	**1.6**	**0.2**	acetyl-coenzyme A synthetase
PA4770	lldP	**-1.7**	**0.8**	L-lactate permease
PA4937	rnr	**-1.5**	**0.2**	exoribonuclease RNase R
PA4983		**1.8**	**0.9**	probable two-component response regulator
PA4985		**1.5**	**0.3**	hypothetical protein
PA5056	phaC1	**1.6**	**0.4**	poly(3-hydroxyalkanoic acid) synthase 1
PA5060	phaF	**-1.5**	**0.2**	polyhydroxyalkanoate synthesis protein PhaF
PA5075		**2.0**	**0.5**	probable permease of ABC transporter
PA5112	estA	**1.7**	**0.6**	esterase EstA
PA5152		**4.2**	**1.0**	probable ATP-binding component of ABC transporter
PA5167		**5.3**	**1.5**	probable c4-dicarboxylate-binding protein

PA5168		**9.7**	**4.5**	probable dicarboxylate transporter
PA5220		**1.5**	**0.5**	hypothetical protein
PA5235	glpT	**1.9**	**0.4**	glycerol-3-phosphate transporter
PA5285		**1.5**	**0.1**	hypothetical protein
PA5301		**2.4**	**0.8**	probable transcriptional regulator
PA5324		**3.8**	**1.4**	probable transcriptional regulator
PA5348		**5.4**	**0.9**	probable DNA-binding protein
PA5442		**3.0**	**1.4**	conserved hypothetical protein
